# The Influence of the Strain Rate on Texture Formation During the Plane Strain Compression of AZ80 Magnesium Alloy

**DOI:** 10.3390/ma17246292

**Published:** 2024-12-23

**Authors:** Yebeen Ji, Jimin Yun, Kibeom Kim, Tae Hee Lee, Kwonhoo Kim

**Affiliations:** 1 Department of Marine Design Convergence Engineering, Pukyong National University, 45 Yongso-ro, Nam-gu, Busan 48513, Republic of Korea; wldpqls@naver.com (Y.J.); dbswlals333@naver.com (J.Y.); 2 Department of Advanced Materials Science, Graduate School of Frontier Sciences, The University of Tokyo, 5-1-5, Kashiwanoha, Kashiwa-shi, Chiba 277-8561, Japan; rlarlqja0874@gmail.com; 3 Premium Vehicle R&D Center, Jeonnam Division, Korea Automotive Technology Institute, 46, F1-ro, Samho-eup, Yeongam-gun, Cheonan 58463, Republic of Korea; 4 Department of Metallurgical Engineering, Pukyong National University, 45 Yongso-ro, Nam-gu, Busan 48513, Republic of Korea

**Keywords:** magnesium alloy, texture, plan strain compression, strain rate

## Abstract

Controlling microstructure and texture development is a key approach to improving the formability of magnesium alloys. In this study, the effects of the strain rate and initial texture on the texture evolution of magnesium alloys during high-temperature processing are investigated. The plane strain compression of three types of AZ80 magnesium alloys with different initial textures was assessed at 723 K and a train rate of 0.0005 s^−1^. Work softening was consistently observed in the stress–strain curves of all samples. However, the peak stress varied depending on the initial texture, with lower peak stress observed under conditions favoring prismatic slip. Under these conditions, the activation of non-basal slip suppressed the formation of basal texture. The texture shifted and developed parallel to the transverse direction when prismatic slip was dominant. In contrast, the activation of pyramidal slip led to the formation of a basal texture tilted by 25° from the (0001) plane. The effects of recrystallization and grain boundary migration on texture development were minimal. This study contributes to understanding the texture development mechanisms in magnesium alloys and provides insights into improving their workability and ductility through texture modification.

## 1. Introduction

Magnesium alloys have significant potential as lightweight structural materials, and these materials are increasingly attracting attention in various fields, such as the automobile industry [1,2,3,4]. However, the processing methods available for magnesium alloys are limited due to their low ductility and formability at room temperature, which result from the restricted number of active slip systems [5,6,7,8,9].

The limited slip systems in magnesium alloys are due to the variable critical resolved shear stress (CRSS) of non-basal slips, such as pyramidal and prismatic slips. The CRSS of basal slip and non-basal slip differs by approximately 1/00 at room temperature and gradually decreases with increasing temperature. Consequently, high-temperature processing is generally more favorable for these alloys. While high-temperature deformation provides sufficient formability for magnesium alloys, it also promotes the development of strong textures due to the low symmetry of their crystal structure. For instance, basal texture forms during the initial stages of hot rolling and compression and intensifies as strain increases. The basal texture limits further plastic processing and leads to anisotropic mechanical properties, thereby necessitating texture control [10,11,12,13,14,15,16].

Despite extensive research on texture control methods for magnesium alloys, further studies are required to fully understand the texture evolution behaviors of hexagonal close-packed (HCP)-structured magnesium alloys, particularly under varying strain rates and temperature conditions. The formation of (0001) basal texture during the high-temperature uniaxial and plane strain compression of Aluminum-Zinc (AZ) series magnesium alloys is primarily attributed to lattice rotation induced by slip and twinning, as well as the preferential growth of grains with specific orientations during dynamic recrystallization (DRX). This preferential grain growth is driven by the stored energy differences between the grains, which become more pronounced under conditions dominated by solute drag effects [5,6,7,9,14].

In face-centered cubic (FCC) and face-centered cubic (FCC) alloys such as Al-Mg and Fe-Si systems, stronger preferential texture development has been reported as solute drag effects intensify [17]. However, in AZ80 magnesium alloys with HCP structure, unique behaviors have been observed under slow strain rates and high-temperature conditions where solute drag effects dominate. Although the (0001) orientation is generally identified as a preferential growth direction, the intensity of the basal texture was observed to weaken progressively under these conditions, accompanied by a 29° rotation of the texture component [6]. These findings are contrary to results observed in FCC and BCC systems, suggesting the involvement of alternative mechanisms specific to HCP structures.

This study aims to investigate the reasons behind the weakening and rotation of basal texture under these conditions and analyze the effects of strain rate and temperature on the texture evolution of AZ80 magnesium alloys. In particular, the experimental conditions of 723 K and a strain rate of 0.0005 s^−^¹ were selected to replicate solute drag-dominated conditions. These conditions also provide an optimal environment to observe unique texture development behaviors that deviate from those reported in previous studies on HCP structures.

By elucidating the mechanisms governing texture evolution under these conditions, this research seeks to highlight the potential for effective texture control through processing parameters alone. Such an approach could complement existing texture control methods and enhance the formability of AZ80 magnesium alloys. Ultimately, these insights may pave the way for broader applications of magnesium alloys in lightweight structural components, particularly in industries where performance optimization and weight reduction are critical.

## 2. Materials and Methods

**Figure 1:**
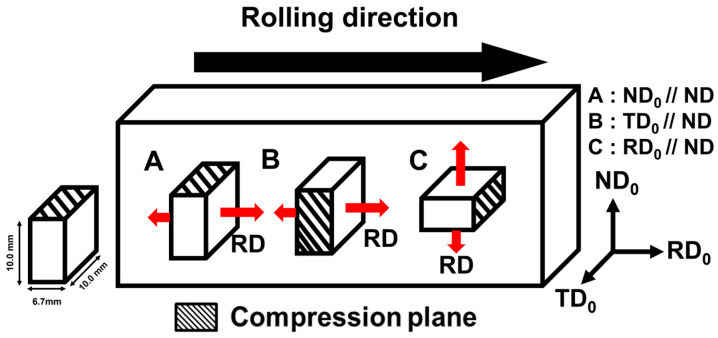
Schematics of specimens: Type A, Type B, and Type C.

**Figure 2:**
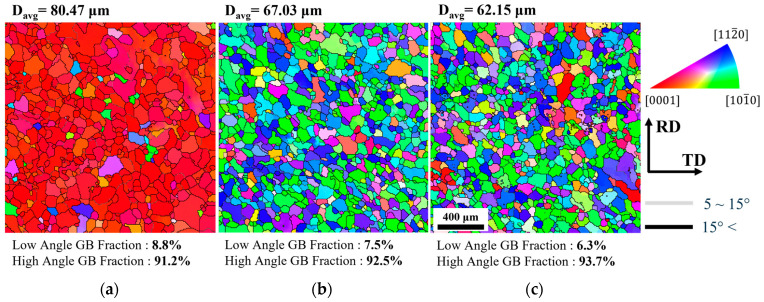
Microstructures of specimens after rolling and annealing: (**a**) Type A, (**b**) Type B, and (**c**) Type C.

In this study, a commercial AZ80 magnesium alloy (Mg-7.98 wt% Al-0.46% Zn-0.16% Mn) was used. Ingots with dimensions of 40 mm × 50 mm × 13.4 mm were heated at 673 K for 1 h and then hot-rolled to a reduction rate of 25%. To prevent crack formation due to abrupt deformation during the rolling process, the reduction rate per pass was limited to 1–5%, and the ingots were reheated at 673 K for 10 min after each pass. After rolling, the material was machined into 6.7 mm × 10 mm × 10 mm specimens to create samples with different initial textures, as shown in Figure 1. In Figure 1, the compression plane is indicated by diagonal lines, and the extension direction during compression is marked with a red arrow. Each specimen was classified as Type A, B, or C based on the compression direction. Specifically, Type A was set with the compression axis parallel to ND_0_ (normal direction), Type B to TD_0_ (transverse direction), and Type C to RD_0_ (rolling direction). Type A, B, and C are specimens with initial textures produced through extrusion, and the results of these can be found in a previous study [5]. To obtain a homogeneous microstructure, each specimen was sealed in a quartz tube filled with argon gas and then heat-treated at 723K for 1 h, followed by water quenching. Figure 2 shows microstructures of the ND plane of each type of specimen after rolling and homogenization heat treatment, where it can be observed that most of the grains were recrystallized as a result of heat treatment.

High-temperature plane strain compression was performed using a universal testing machine. The specimens were mounted in a channel die to restrict extension in the TD and compressed at 723 K with a strain rate of 0.0005 s^−1^ up to true strains of −0.4, −0.7, and −1.0, respectively. Immediately after plane strain compression, water quenching was performed to prevent microstructural changes due to the residual heat. The compressed specimens were then cut parallel to the compression plane (ND plane), and textures and the microstructures on the central region were observed.

**Figure 3:**
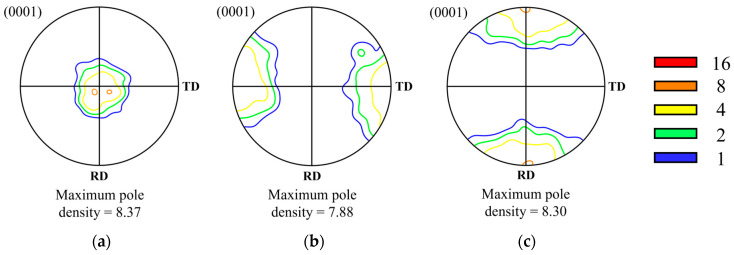
The (0001) pole figures of (**a**) Type A, (**b**) Type B, and (**c**) Type C specimens before plane strain compression.

The samples were prepared through mechanical polishing and electrolytic polishing. Electrolytic polishing was conducted using a 2–4% perchloric acid solution mixed with 99.9% ethanol, with the voltage adjusted to achieve a current density of 0.20–0.22 A/cm² at 243K. Subsequently, microstructure and texture observations were performed using an FE-SEM (field-emission scanning electron microscopy) device (CLARA, TESCAN, Brno, Czech Republic) and EBSD (electron backscatter diffraction; Symmetry S2, Oxford Instruments, Abingdon, UK). EBSD measurements were taken over an area of 1800 μm × 1800 μm with a step size of 4 μm. The raw data were analyzed using TSL (OIM Analysis 7) and ATEX (version 4.14) software. Approximately 200,000 points were examined in each image, and only datasets with an initial indexing rate of over 90% were utilized. The orientation distribution function (ODF) was calculated using the harmonic series expansion method. Figure 3 shows the (0001) pole figures for the three types of specimens before compression. The pole density is represented as multiples of the average pole density, with contours indicating these multiples. Figure 3a shows the typical rolling texture of magnesium alloys, while Figure 3b,c demonstrate the formation of textures in the TD and RD, respectively. These results indicated different initial textures in three kinds of specimens.

## 3. Results

### 3.1. True Stress–Strain Curves

**Figure 4:**
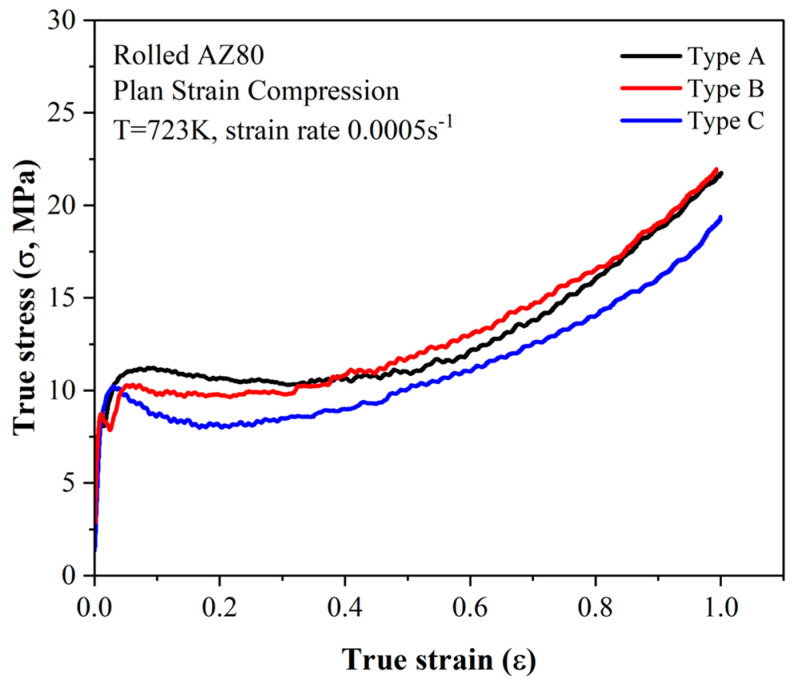
True stress–strain curves for deformation at 723 K up to a strain of −1.0 at a strain rate of 0.0005 s^−1^.

Figure 4 shows the true stress–strain curves obtained from the plane strain compression tests. In this figure, the true strain is expressed as an absolute value. All specimens exhibited a rapid increment in stress during the initial stage of deformation, followed by a decrease in stress due to work softening. The flow stress remained constant regardless of the increase in strain but began to rise again after approximately a strain of −0.4. There were slight differences in the initial peak stress and the subsequent flow stress depending on the initial texture of the specimens. The peak stress was highest in the Type A specimen, followed by Types C and B. After work softening, the flow stress was nearly identical for the Type A and B specimens, while the Type C specimen maintained a generally lower flow stress up to a strain of −1.0. These characteristics on the stress curves were also observed in the previous studies using AZ61, AZ80, and AZ91 alloys. Additionally, this behavior became more obvious when the strain rate was increased [7,9,14].

### 3.2. Texture Formation Behavior During the Deformation

#### 3.2.1. Type A

**Figure 5:**
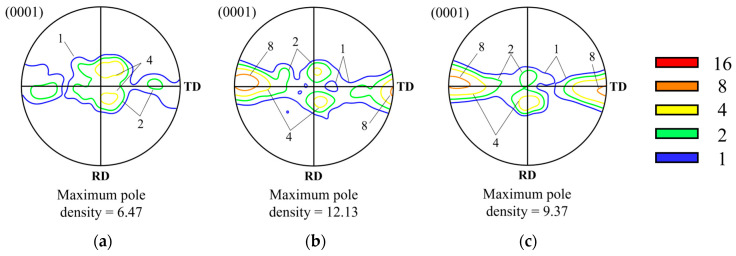
The (0001) pole figures of Type A specimens compressed at 723 K and 0.0005s^−^¹ after true strains of (**a**) −0.4, (**b**) −0.7, and (**c**) −1.0.

Figure 5 shows the (0001) pole figures observed after compressing the Type A specimen to true strains of (a) −0.4, (b) −0.7, and (c) −1.0 respectively. The maximum pole density formed under each condition is also indicated on each pole figure. The initial texture of the Type A specimen before compression was found to be the typical rolling texture of magnesium alloys. During the early stages of deformation, the basal texture tilted by 25° along the ND-RD, while a gradual splitting in the ND-TD was observed. This texture evolution is similar to that observed in AZ-series magnesium alloys under plane strain compression at a strain rate of 0.05 s^−1^, except for the tilting of the basal planes toward the TD. The components formed during the initial stages of deformation remained stable throughout the compression process [7,9]. The pole density of the ND-RD component decreased from 4 to 2 within the 20° to 25° range. In contrast, the texture splitting toward the ND-TD was gradually intensified, eventually forming a maximum pole density of 9.37 in the TD.

#### 3.2.2. Type B

**Figure 6:**
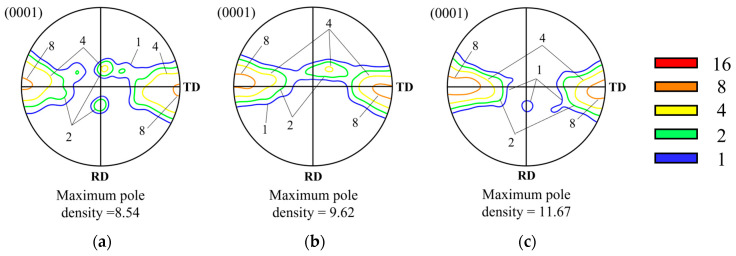
The (0001) pole figures of Type B specimens compressed at 723 K and 0.0005s^−^¹ after true strains of (**a**) −0.4, (**b**) −0.7, and (**c**) −1.0.

Figure 6 shows the (0001) pole figures for the Type B specimen. Under the compression conditions, the main texture component remained largely unchanged from the initial texture, except for a slight elongation and splitting between the ND and RD, similar to Type A. During the deformation process, a slight formation of basal components tilted toward the ND-TD was observed, but these components nearly disappeared by the time a strain of −1.0 was reached. The maximum pole density appeared in the TD both before and after deformation, increasing to 11.67 after the compression was completed.

#### 3.2.3. Type C

**Figure 7:**
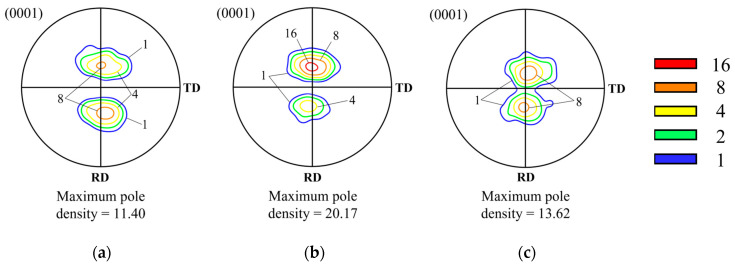
The (0001) pole figures of Type C specimens compressed at 723 K and 0.0005 s ^−1^ after true strains of (**a**) −0.4, (**b**) −0.7, and (**c**) −1.0.

Figure 7 shows the (0001) pole figures for the Type C specimen. Unlike Types A and B, the main texture component under these conditions was observed to be tilted in the ND-TD. The texture initially formed in the TD gradually shifted toward the ND with deformation, ultimately forming a major component around 20°. The maximum pole density reached its peak at the strain of −0.7, with a value of 20.17, but decreased to 13.62 when the true strain reached −1.0.

### 3.3. Microstructural Development

**Figure 8:**
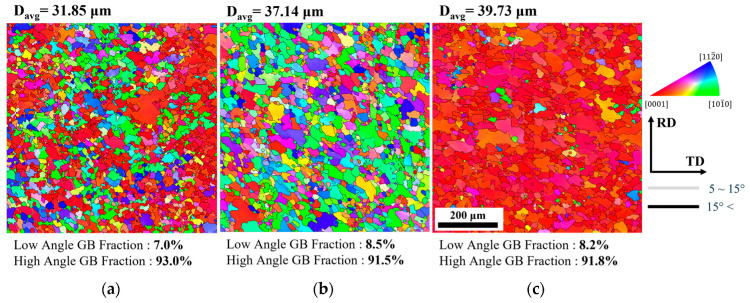
Microstructures of (**a**) Type A, (**b**) Type B, and (**c**) Type C specimens according to EBSD measurements at a strain rate of 0.0005 s^−^¹ after deformation up to a strain of −1.0.

Figure 8 shows the microstructures of each type of specimen after compression to a strain of −1.0. The microstructures were captured on the ND plane using SEM-EBSD. Each grain is represented based on the misorientation between points measured by EBSD. High-angle grain boundaries (HAGBs) with misorientations greater than 15° are depicted with black lines, while low-angle grain boundaries (LAGBs) with misorientations between 5° and 15° are shown with gray lines. The orientation of each grain is indicated using colors corresponding to the standard inverse pole figure triangle. All three types exhibited significant grain refinement due to dynamic recrystallization occurring during the deformation process. Most recrystallized grains were found to form and grow near the grain boundaries, and the orientations of these recrystallized grains mostly followed those of the parent grains. Additionally, coarse grains were still present in the microstructure, and these grains exhibited growth regardless of the extension direction.

**Figure 9:**
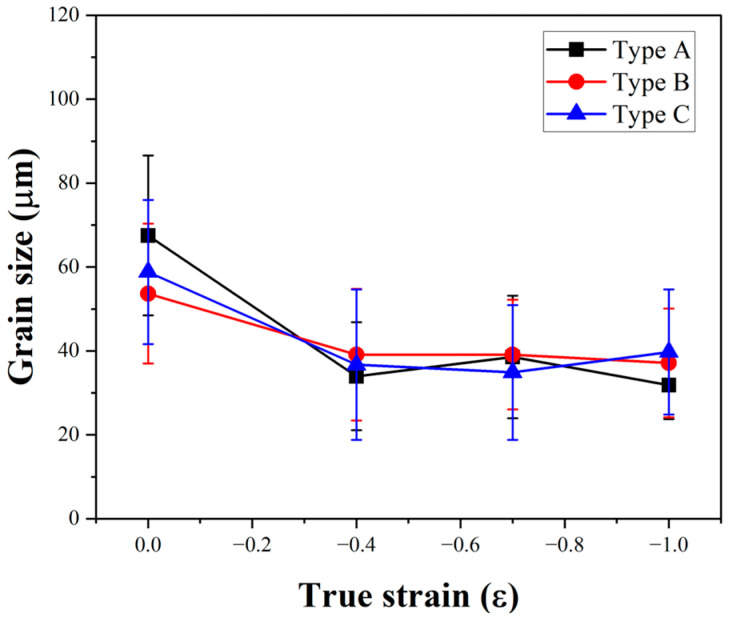
The average grain size of each type specimen after plane strain compression from strain 0 to −1.0.

Figure 9 presents the average grain size and standard deviation values observed at each strain level. At a strain of −0.4, the average grain size of all three types decreased to a range of 33–39 µm, with no further significant reduction observed. The wide range of standard deviations observed under each condition is attributed to the presence of coarse grains, as shown in Figure 8. While the standard deviation gradually decreased with increasing strain, a wide distribution remained even at a strain of −1.0. Furthermore, the Type C specimen exhibited the widest standard deviation among all strain levels.

**Figure 10:**
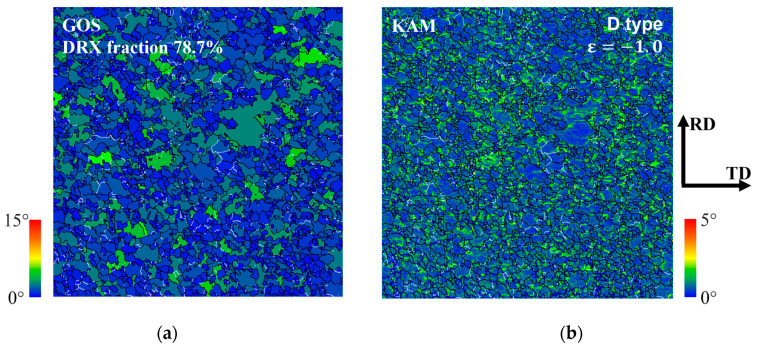
(**a**) GOS and (**b**) KAM map of the Type A specimen observed after the plane strain compression, with a strain of up to −1.0.

Figure 10 shows the (a) grain orientation spread (GOS) map and (b) kernel average misorientation (KAM) map of the microstructure observed in the Type A specimen from Figure 8. GOS represents the average orientation deviation within individual grains, while KAM indicates the average local misorientation between a measured point and its neighboring points. GOS was observed to assess the occurrence of recrystallization, with grains with a GOS value of less than 2° defined as recrystallized grains. As shown in Figure 10a, the fraction of recrystallized grains in the Type A specimen was 88.3%, confirming a microstructure consisting of a mixture of recrystallized and deformed grains. Grains with relatively high accumulated misorientation were mostly coarse and irregularly shaped grains, as observed in Figure 8. In Figure 10b, misorientations within these grains were evenly distributed throughout the ingrains. Although in Figure 10, the recrystallized grain fractions for Types B and C were 88.3% and 77.8%, respectively, and similar misorientation distributions were observed. These characteristics of microstructural development suggest that dislocation movement occurred under solute drag conditions at this strain rate. This will be discussed in more detail below.

## 4. Discussion

### 4.1. Deformation Behaviors and Mechanisms

In this study, plane strain compression was analyzed at a temperature of 723 K and a strain rate of 0.0005 s^−1^ to investigate the effect of initial texture on microstructure and texture development behavior. The results showed that Types A, B, and C exhibited different texture characteristics: Type A had a mixed texture with basal components tilted toward the RD and split in the TD, Type B displayed texture with only basal components split in the TD, and Type C showed texture with only basal components tilted toward the RD. A common feature among all three types, however, was the suppression of basal texture development, which is typically observed during compression processes. These results differ from previous studies where the compression was conducted under the strain rate of 0.05 s^−1^, showing the development of the basal texture components regardless of the initial texture [5,6,7,14].

The suppression of basal texture is significant because it often hinders the activation of basal slip—essential for accommodating deformation—and prismatic slip, which plays a critical role in subsequent deformation, at both room and high temperatures. In this study, the reduced CRSS difference between basal and non-basal slip systems at a strain rate of 0.0005 s^−^¹ likely facilitated the activation of non-basal slip, disrupting the conditions required for basal texture growth. Unlike at higher strain rates, where basal texture dominates, the observed tilted basal texture facilitated the sustained activation of both slip systems. Additionally, the prismatic texture that developed through this process enabled the continuous operation of prismatic slip, overcoming the restrictions imposed by conventional basal textures. This finding underscores the importance of processing parameters in tailoring texture evolution and improving the formability of AZ80 magnesium alloys. These findings suggest that the strain rate and initial texture significantly influence the interplay between slip systems, dislocation movement mechanisms, and recrystallization behavior, ultimately governing texture evolution in AZ80 magnesium alloys. By disrupting basal texture dominance, these processing conditions provide a pathway for enhancing workability through controlled texture evolution. To apply and utilize this in practice, it is necessary to investigate the mechanisms through which these factors operate. Therefore, this section explores these mechanisms in detail.

In magnesium alloys, twinning, alongside slip, acts as a significant deformation mechanism, primarily due to the limited slip systems available in the HCP crystal structure caused by its asymmetry. Non-basal slip, in particular, has a temperature-dependent CRSS, making twinning more active at room temperature, where it has a relatively lower CRSS [8]. Twinning can still occur even at sufficiently high temperatures where non-basal slip is active [18,19]. Twinning is preferred when the compression axis is perpendicular to the c-axis, rotating the c-axis by approximately 86°, aligning it closer to the ND. In this context, the basal components observed near the ND in Figure 6a and Figure 7a may have been formed by $\{10\bar{1}2$} twinning. However, direct evidence of twinning was not observed in the microstructure or misorientation distributions; thus, twinning was not considered in this study.

The activation of slip systems in each specimen, depending on the initial texture, varies according to the Schmid factor and geometric relationships. In this study, all specimens were subjected to conditions. The angle between the c-axis and the compression axis was either parallel (Type A) or perpendicular (Types B and C) before deformation. This restricted basal <a> slip in all cases during the early stages of deformation. From the same perspective, Type A has difficulty activating prismatic <a> slip, so deformation is mainly accommodated by pyramidal <c+a> slip. In contrast, both prismatic <a> slip and pyramidal <c+a> slip could be activated in Types B and C. However, due to the geometric constraints, slip along the c-axis is more restricted in Type B. This restriction leads to the preferential activation of prismatic <a> slip. Meanwhile, in Type C, slip systems along the a-axis are more restricted, resulting in the preferential activation of pyramidal <c+a> slip systems during the initial stages of deformation.

Previous studies on AZ80 and AZ91 magnesium alloys under a strain rate of 0.05 s^−1^ have shown that different peak stresses are formed depending on the initial texture [5,14]. However, in Figure 4, it is observed that plane strain compression at a strain rate of 0.0005 s^−1^ results in nearly identical peak stresses regardless of the initial texture. As explained earlier, and consistent with previous studies, the three types of specimens used in this study all experienced restricted basal <a> slip. Either prismatic <a> slip or pyramidal <c+a> slip was preferentially activated, depending on the texture components. Under a strain rate of 0.05 s^−1^, Types A and C, which favored pyramidal <c+a> slip activation, exhibited higher peak stresses compared to Type B, which favored prismatic <a> slip activation. This difference is likely related to the CRSS of the two non-basal slip systems. It is well known that, unlike basal <a> slip and twinning, the CRSS of non-basal slip is sensitive to temperature variations. Prismatic <a> slip generally has a lower CRSS than pyramidal <c+a> slip across most temperatures [8,15]. Additionally, the tendency of <c+a> slip to easily separate into <a> and <c> dislocations can increase the work hardening rate, potentially leading to higher peak stress even if the CRSS values are similar [13,20]. Furthermore, the reduced difference in peak stress among the three types at a strain rate of 0.0005 s^−1^ suggests that the CRSS of non-basal slip may also vary with strain rate, in addition to deformation temperature. This observation aligns with the suggestions of Ulacia et al. They reported that in uniaxial compression of AZ31 magnesium alloy, conditions favoring the activation of extension twinning resulted in similar yield stresses, regardless of the temperature and strain rate. In contrast, conditions favoring the activation of non-basal slip showed yield stress variations depending on both the temperature and strain rate [18]. While Ulacia’s results indicated that the CRSS of pyramidal <c+a> slip is more influenced by the temperature than the strain rate, they also demonstrated that yield stress changes more significantly with the strain rate at processing conditions above 200 °C. This suggests that the influence of the strain rate increases at higher temperatures.

These findings confirm that strain rate and initial texture significantly affect slip system activation and peak stress behaviors, aligning with the influence of non-basal slip systems at elevated temperatures. Although no direct evidence was obtained in this study, the observed behaviors, such as nearly identical peak stresses across different textures and the tilted basal texture, suggest that pyramidal <c+a> slip activation becomes more prominent under slower strain rates [8,18]. However, further experimental verification, such as transmission electron microscopy (TEM) to observe dislocation structures, is necessary to directly confirm this conclusion.

### 4.2. Behaviors of Texture Formation

The formation and development of texture during the high-temperature processing of magnesium alloys are influenced by the combination of active slip systems and dynamic recrystallization. In AZ-series magnesium alloys, it is common to form a basal texture during rolling or compression at room or high temperatures, where the processing axis and the c-axis align parallel to each other [5,6,7,13,15,16,18]. This phenomenon is primarily observed when basal <a> slip is the dominant deformation mechanism. The basal texture becomes sharpened as non-basal slip becomes more restricted. Prismatic <a> slip and pyramidal <c+a> slip are known to induce lattice rotation of the basal planes in the RD and TD, respectively, and the activation of non-basal slip tends to inhibit the development of a strong basal texture [15,18,21]. In this context, Hadorn et al. reported that the addition of rare earth (RE) elements reduces the CRSS of prismatic <a> slip, thereby weakening the basal texture [22].

During the high-temperature uniaxial and plane strain compression of AZ80 magnesium alloy at a strain rate of 0.05 s^−1^, the formation of a basal texture was observed in all specimens [5,6,7]. This suggests that basal <a> slip remained the primary deformation mechanism at this strain rate. Furthermore, under conditions similar to those of the Type A specimen, the angular relationship caused the Schmid factor values for other slip systems to approach zero, leading to the preferential activation of pyramidal <c+a> slip. However, the lattice rotation toward the RD did not occur, and the basal texture remained aligned with the ND. This suggests that texture formation is not solely governed by slip-induced lattice rotation but may also be influenced by the preferential formation and growth of grains oriented in the (0001) direction through continuous dynamic recrystallization [6].

When the strain rate decreased from 0.05 s^−^¹ to 0.0005 s^−^¹, the microstructure showed a reduction in the fraction of DRXed grains and coarse, irregularly shaped grains [7]. These coarse grains exhibited elongated shapes or even connected with other grains, regardless of the RD or TD, which reflects a transition in the dislocation movement mechanism from dislocation glide to solute drag. Previous studies have suggested that this transition can be predicted through the stress exponent (n) value, with n = 5 indicating dislocation glide and n = 3 indicating solute drag, depending on the processing temperature and strain rate [6]. In addition, they observed that in the uniaxial compression of AZ80 alloy, the dislocation movement mechanism shifted when the peak stress was around 20 MPa, while in this study, all three types exhibited peak stresses around 10 MPa.

When the dislocation movement mechanism was dominated by solute drag, solute atoms partially alleviated the stress field and reduced the dislocation velocity, leading to a more uniform distribution of dislocations within the grains. In this scenario, the accumulation of dislocations was limited, making it difficult to reach the driving force required for recrystallization. As a result, the grains exhibited different deformation energies depending on the lattice components, with the difference in deformation energy increasing with strain and promoting grain boundary migration. This feature is observed in Figure 8 and Figure 10.

At lower strain rates, such as 0.0005 s^−^¹, the CRSS difference between basal slip and non-basal slip became negligible, allowing non-basal slip systems, particularly pyramidal <c+a> slip, to activate more readily. In contrast, at 0.05 s^−^¹, basal slip dominated the deformation process, leading to limited lattice rotation and the stabilization of basal texture. However, at 0.0005 s^−^¹, the enhanced activation of non-basal slip promoted significant lattice rotation, violating the stability condition required for the preferential growth of grains during grain boundary migration. Specifically, for grains to grow preferentially, their orientation must remain stable against lattice rotation. This condition is disrupted at lower strain rates, leading to the weakening of basal texture. Experimental results confirm this, as the basal texture was tilted by approximately 15–20° toward the RD, and its intensity decreased with increasing strain. These observations suggest that texture development is more influenced by slip-induced lattice rotation than by grain boundary migration.

**Figure 11:**
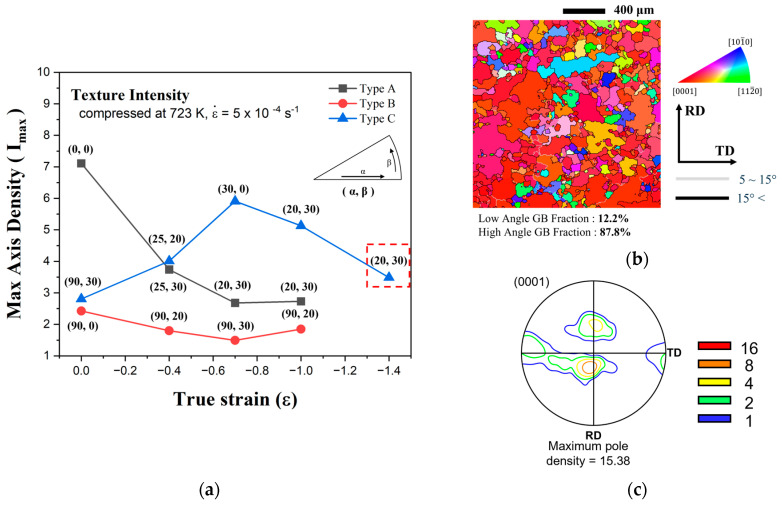
(**a**) A relationship between texture intensities and true strain. Texture components are shown above each point. (**b**) Microstructure and (**c**) the (0001) pole figure of Type C specimen compressed up to the true strain −1.4.

Figure 11a shows the principal components and axis densities of the inverse pole figures (IPF) observed after compression for each type at true strains ranging from 0 to −1.4. The principal components are represented as coordinates (α, β), where α indicates the angle between [0001] and $\left[10\bar{1}0\right]$, and β represents the angle between $\left[10\bar{1}0\right]$ and $\left[11\bar{2}0\right]$. Figure 11b,c show the microstructure and the (0001) pole figure for Type C at a strain of −1.4. The IPF characteristics in Figure 11a are largely consistent with the texture evolution behaviors of each type observed in Figure 5, Figure 6 and Figure 7. For Types A and B, the axis densities decreased or remained constant with increasing strain, with principal components located at approximately (20, 30) or (90, 20). Similarly, Type C exhibited comparable principal components; however, its axis density increased up to a strain of −0.7 and subsequently decreased.

The decrease in axis density after strain −0.7 for Type C aligned with the trend observed in the other types, where the c-axis gradually rotated toward the TD. This behavior is also evident in Figure 11c. One notable observation from Figure 11b is that Type C shows more pronounced grain growth compared to that at previous strain levels. This enhanced grain growth, driven by grain boundary migration, is attributed to the increasing differences in deformation energy with increasing strain. Nevertheless, the gradual shift in the texture toward the TD supports the notion that texture development is predominantly influenced by slip-induced lattice rotation.

The results from this study indicate that during the plane strain compression of AZ80 magnesium alloy, the activation of non-basal slip increased significantly as the strain rate decreased from 0.05 s^−1^ to 0.0005 s^−1^. This increase had a substantial impact on texture development behaviors. Li et al. used a grain interaction model in their simulation to predict changes in texture development during plane strain compression as the activation of non-basal slip varied [23]. According to their results, in an initial texture similar to Type C, the CRSS ratios for slip systems and twinning were as follows: basal <a>: prismatic <a>: pyramidal <a>: pyramidal <c+a>: tension twinning at 1:7:8:9:12. The combination of pyramidal <c+a> and prismatic <a> slip in the early stages of deformation randomized the texture. This was followed by the activation of basal <a> slip, which gradually rotated the texture toward the ND. This result likely explains the reason why the flow stress of Type C is lower than that of the other types in Figure 4, as it may be related to the high activation of basal <a> slip. In the initial texture of Type B, when the activation <a> slip was dominant, the texture gradually rotated from the TD to ND. However, when the activation of prismatic <a> slip was similar to or higher than that of basal <a>, the c-axis remained parallel to the TD, and the $\left(10\bar{1}0\right)$ and $\left(11\bar{2}0\right)$ planes rotated toward the RD. This aligns well with the observation that the texture intensity of Type B was the lowest under all strains, as shown in Figure 11. The continued rotation of the prismatic plane when the c-axis is parallel to the TD has been confirmed by Park et al. [24].

## 5. Conclusions

To investigate the effect of strain rate variation on the microstructure and texture development behavior in AZ80 magnesium alloy, a plane strain compression test was performed at 723 K and a strain rate of 0.0005 s^−1^ on three types of specimens with different initial textures. The results were then compared with the data from previous studies. The conclusions are as follows:

1. At a strain rate of 0.0005 s^−^¹, the CRSS of non-basal slip systems was observed to decrease significantly, reducing the CRSS ratio between basal and non-basal slip systems. This narrowing of the CRSS gap resulted in nearly identical peak stresses of approximately 10 MPa across all initial texture types, as observed in the true stress–strain curves.
2. Under slow strain rate conditions, solute drag was the dominant deformation mechanism. Dynamic recrystallization was weakened, and grain boundary migration became more pronounced. However, the grain boundary migration hardly affected texture development, as texture evolution was primarily governed by slip-induced lattice rotation.
3. The activation of non-basal slip systems, particularly prismatic and pyramidal <c+a> slip, inhibited the development of basal texture, tilting it by approximately 20° toward the RD. The continued activation of prismatic slip promoted the development of prismatic texture, with the c-axis aligned parallel to the TD. This represents a significant deviation from the basal-dominated texture observed at higher strain rates.
4. These findings emphasize the potential for advanced texture control in AZ80 magnesium alloys by integrating processing parameter adjustments with existing control strategies. The suppression of basal texture as well as the enhancement of prismatic texture under slow strain rate conditions offer a robust framework for tailoring the mechanical properties of magnesium alloys. This knowledge provides a pathway for optimizing forming processes in industries requiring lightweight materials, such as automotive and aerospace sectors, where improved formability and reduced anisotropy are critical.

## Data Availability

The original contributions presented in this study are included in the article. Further inquiries can be directed to the corresponding authors.
